# Ex-Confederates Disfranchised

**Published:** 1894-01

**Authors:** 


### EDITORIAL DEPARTMENT.

F. E. DANIEL, M. D., Editor.
S. E. HUDSON, M. D., Managing Editor.
A. J. SMITH. M. D., Galveston, Associate Editor.

EDITORIAL STAFF:

PROF. J. E. THOMPSON, M. D., Texas Medical College, Galveston; Surgery.
PROF. WM. KEILLER, M. D., Texas Medical College, Galveston; Obstetrics and Gynecology.
PROF. DAVID CERNA, M. D., Texas Medical College, Galveston; Therapeutics.
PROF. A. J. SMITH, M. D., Texas Medical College, Galveston; Medicine
DR. ISADORE DYER, Tulane University; Dermatology.
DR. R. H. L. BIBB, Saltillo, Mexico; Foreign Correspondent.
DR. ROBT. MORRIS, Charity Hospital, N. O.; Clinical Reports

———o———

The JOURNAL is the official organ of the Austin District Medical Society, the West Texas District Medical Society and the Galveston County Medical Society.

### EX-CONFEDERATES DISFRANCHISED.

In our last issue we had an editorial on the subject of Commissioner Lochren's "ruling" that ex-Confederate soldiers or surgeons should not hold the position of medical examiner on any U. S. pension board, and we entered a protest against it, as arbitrary and unjust. Since that time an Austin physician, a distinguished ex-Confederate, has received a letter from a Texas congressman, requesting him to recommend for appointment on the board, at Austin, as medical examiner, a physician who was not disqualified (as was this physician himself) by reason of a Confederate record. (That is the purport of the letter, if not the wording.) Hence, there can be no doubt that such an order, or "ruling," has been promulgated. A well-known Confederate surgeon, residing in Austin, made application for the position, and his application bore such endorsement as would, in the absence of said "ruling," undoubtedly have secured the appointment; in fact he had been notified of his appointment, but not commissioned; it was reconsidered. So here are two cases in point where the disfranchisement has applied. It was claimed by certain friends of the administration that we were premature; that it was only newspaper report that such action had been taken; and no wonder,it is so monstrous that they, and the administration, may well be ashamed of it.

The right to hold office is one of those "vested rights" of citizens guaranteed by the constitution; and while we suppose there is no law to compel the commissioner to appoint an ex-Confederate,—he can be as arbitrary as he pleases in the selection of his appointees,—no court of equity would sustain him in removing a medical examiner for this cause alone. The most astonishing thing, in connection with it, is the fact that the President, whom we had always regarded as the embodiment of fair play and justice, should consent to the enforcement of a "ruling" which practically disfranchises every one who was voluntarily, or involuntarily, connected with the Confederate army.

It has been said, in attempting to justify, or palliate, the action of the commissioner, that the present administration suspects that there have been frauds practiced on the pension bureau, and that a thorough overhauling will be made Every pensioner will be re-examined; and the administration does not want the Republicans to be able to say that the examinations were made by the "recent enemy's" surgeons! Worse and worse. That is to say, that a physician who had been in the Confederate army, in any capacity, it may be, as a private, when a *boy*, or, it may be, as a surgeon, now gray-haired and venerable, could not or would not do justice to an applicant for pension under the law! It is a stigma, an insult to the entire medical profession. Were the pension boards all over the United States to be composed entirely of ex-Confederates, the inference would not be justifiable; but in any event, under the most liberal distribution of these offices amongst ex-Confederates, that any of us could wish, how large a per cent., or, rather, how small a per cent. would be of the ex-Confederate class. It is a mere pretext; a contemptible subterfuge.

The Austin District Medical Association met in this city on the 21st of December, ult., and the subject being brought up, the following resolutions were unanimously adopted. The subject has also been brought to the attention of other local societies, and they will undoubtedly take action on it; and as it will be seen by the resolution, the State Medical Association will be asked to consider it. The subject will be agitated, and we indulge the hope that "our distinguished President" may be brought to look into it; if so, his sense of justice will surely assert itself, and we may look to have the obnoxious thing rescinded. It could only have been conceived by a very narrow and prejudiced mind, and ten to one, it originated with a person who was never within danger of Confederate bullets, nor within smelling distance of burnt powder:

